# Factors Influencing Residual Glandular Breast Tissue after Risk-Reducing Mastectomy in Genetically Predisposed Individuals Detected by MRI Mammography

**DOI:** 10.3390/cancers15030829

**Published:** 2023-01-29

**Authors:** Frederic Dietzel, Leoni Kolberg, Anne Sophie Vesper, Jürgen Hoffmann, Carolin Nestle-Krämling, Karin Zwiefel, Verena Friebe, Lino M. Sawicki, Nils Martin Bruckmann, Kai Jannusch, Janna Morawitz, Gerald Antoch, Tanja Natascha Fehm, Julian Kirchner, Svjetlana Mohrmann

**Affiliations:** 1 Department of Diagnostic and Interventional Radiology, Medical Faculty, University Dusseldorf, 40225 Dusseldorf, Germany; 2 Department of Obstetrics and Gynecology, Medical Faculty, University Dusseldorf, 40225 Dusseldorf, Germany; 3 Department of Obstetrics and Gynecology, Agaplesion Bethesda Krankenhaus Wuppertal, 42109 Wuppertal, Germany; 4 Department of Gynecology, Evangelisches Krankenhaus Düsseldorf, 40217 Düsseldorf, Germany; 5 Breast Center, Kliniken der Stadt Köln, 51067 Köln, Germany

**Keywords:** magnetic resonance imaging—MRI, breast MRI, MRI mammography, risk-reducing mastectomy, residual glandular tissue, high-risk breast cancer collective

## Abstract

**Simple Summary:**

Residual glandular tissue (RGT) after risk-reducing mastectomy in patients with a familial predisposition (high-risk collective) is associated with a risk of breast cancer that cannot be precisely determined. The residual risk should be as assessable as possible. For this purpose, a monocentric retrospective analysis of measurable factors influencing the postoperative residual glandular tissue was performed. Analyzed were 117 breasts, 63 left and 54 right, from a cohort of 81 patients (carriers of a pathogenic mutation) with unilateral (36 patients) or bilateral (45 patients) risk-reducing mastectomy. Consultation and possible testing were performed with the patient’s consent at the FBREK (Familial Breast and Ovarian Cancer) Center of the University Hospital Düsseldorf (UKD), or in advance at another site of the German Consortium for Familial Breast and Ovarian Cancer. MRI-assisted detection of residual skin flap thickness and volumetry of each breast were performed. Residual glandular tissue was recorded volumetrically. In addition, patient-related covariates were recorded, and their influence on postoperative residual glandular tissue and skin flap thickness was analyzed using univariate and multivariate regression.

**Abstract:**

Purpose: This study seeks to evaluate MR imaging morphological factors and other covariates that influence the presence of residual glandular tissue after risk-reducing mastectomy in patients with a familial predisposition. Methods: We analyzed women of a high-risk collective with pathogenic mutation (BRCA1 (n = 49), BRCA2 (n = 24), or further mutation (n = 9)). A total of 117 breasts were analyzed, 63 left and 54 right, from a cohort of 81 patients, who were on average 40 years old. The mean follow-up was 63 months (range 12–180 months, SD = 39.67). Retrospective analysis of MR imaging data from 2006–2022 of patients of a high-risk collective (all carriers of a pathogenic mutation) with contralateral (RRCM) or bilateral risk-reducing mastectomy (RRBM) was performed. In the image data the remaining skin flap thickness by distance measurements at eight equally distributed, clockwise points and the retromamillary area, as well as by volumetry of each breast, was elected. Residual glandular tissue was also volumetrized. In addition, patient-related covariates were recorded and their influence on postoperative residual glandular tissue and skin flap thickness was analyzed by uni- and multivariate regressions. Results: A significant association with postoperative residual glandular tissue was shown in multivariate analysis for the independent variables breast density, skin flap mean, and surgical method (all *p*-values < 0.01). A negatively significant association could be seen for the variables preoperative breast volume (*p*-values < 0.01) and surgeon experience (most *p*-values < 0.05–<0.1). Conclusion: Postoperative residual glandular tissue is an important tool for quantifying the risk of developing breast cancer after risk-reducing mastectomy. Different effects on residual glandular tissue were shown for the independent variables breast density, skin flap, surgical method, preoperative breast volume, and surgeon experience, so these should be considered in future surgical procedures preoperatively as well as postoperatively. Breast MRI has proven to be a suitable method to analyze the skin flap as well as the RGT.

## 1. Introduction

Breast cancer is the most common solid tumor in women worldwide, with an annual incidence of approximately 2.3 million new cases per year [1]. The risk in the population of women of developing breast cancer during their lifetime is approximately 12.5%. Similarly, breast cancer is the most common cause of cancer death in women in Germany [2]. In about 30% of women suffering from breast cancer, a familial clustering can be detected and, if a familial predisposition is suspected, genetic analysis can be followed up [3,4]. A mutation in the genes breast-cancer-1 (BRCA1) or breast-cancer-2 (BRCA2) is carried by 25% of patients with familial predisposition [5,6]. If a BRCA1 or BRCA2 mutation is present, affected individuals develop breast cancer about 20 years earlier than women with sporadic cancer, with a cumulative lifetime risk of 60% on average for developing breast cancer and 16–55% for developing ovarian cancer [7]. To achieve lifelong and most extensive risk reduction with confirmed BRCA1/2 mutation, as well as some other high-risk genes, patients have the option of bilateral risk-reducing mastectomy with primary or secondary reconstruction [8,9,10]. Regardless of the surgical technique of mastectomy, residual glandular tissue is reported [11]. A former study has even shown that potentially more residual glandular tissue remains in the breast after risk-reducing prophylactic mastectomy compared with oncologic mastectomy [12]. Residual glandular tissue after risk-reducing mastectomy is associated with a risk of breast cancer that cannot be precisely determined [13,14]. Since these patients are no longer included in intensified screening programs, the residual risk should be assessable as best as possible [11,12]. Thus, the aim of this study is to identify significant effects on postoperative residual glandular tissue and skin flap thickness and to evaluate if they are modifiable or predictable.

## 2. Materials and Methods

This study was approved by the local ethics committee of Heinrich Heine University, Düsseldorf (study number 2021-1462), in conformance with the Declaration of Helsinki [15]. A written informed consent form was obtained from all patients. All MR image data and medical history information processed in the present retrospective, non-interventional study were collected as part of routine clinical practice in the Department of Obstetrics and Gynecology at the University Hospital Düsseldorf and were necessary for the treatment of the patients. All participating surgeons were certified by the German Working Group for Aesthetic, Plastic and Restorative Surgical Procedures in Gynecology e.V. (AWOgyn), a Section of the German Society for Gynecology and Obstetrics (DGGG). The breast MR images were obtained between April 2006 and February 2022. Inclusion criteria were high-risk patients with unilateral or bilateral risk-reducing mastectomy with simultaneous implant insertion. The high-risk collective is defined by the criteria of the German Consortium for Familial Breast and Ovarian Cancer. Only patients who received a recommendation for risk-reducing mastectomy based on their pathogenic mutation and associated increase in risk for developing breast carcinoma were included in the study. Thus, the gene mutations present in our study do not reflect all eligible risk genes. Exclusion criteria were patients with therapeutic mastectomies and patients without familial predisposition. Furthermore, at least one postoperative breast MRI had to be available for each patient. To identify study-relevant patients, the implant catalog of the Interdisciplinary Breast Center and FBREK (Familial Breast and Ovarian Cancer) of the Department of Gynecology at the University Hospital Düsseldorf was used and searched for patients with unilateral or bilateral subcutaneous mastectomy with simultaneous implant insertion. Subsequently, a comparison of the selected patients with the database of the University Hospital Düsseldorf took place in order to filter out those who had received a unilateral or bilateral risk-reducing mastectomy. The final cohort consisted of 81 patients who had shown to have a familial breast cancer predisposition on previous genetic testing and who had also received a RRBM or RRCM with simultaneous implant reconstruction. A total of 72 breasts underwent surgery as RRBM, and 45 underwent surgery as RRCM due to contralateral breast cancer.

Furthermore, the following anamnestic as well as clinical data were collected:

- Age of the patient at the time of surgery;
- Analyzed breast side;
- RRBM or RRCM;
- Follow-up period;
- Premature end of the follow-up period;
- Date of birth;
- Date of surgery;
- Surgeon;
- Surgical method;
- Follow-up operations;
- Previous operations;
- Mutation type.

### 2.1. Study Cohort

In the course of the study, 81 women who fulfilled all necessary inclusion criteria were examined between April 2006 and February 2022. Of the patients, 36 (44.4%) were included in the analysis because they had undergone an RRBM, and 45 patients (55.6%) had undergone an RRCM for breast carcinoma on one side, resulting in a total of 117 breasts, of which 63 (53.8%) were left and 54 (46.2%) were right. Table 1 shows the key data of the study cohort. The mean age of the patients at the time of surgery was 40.12 years (SD 8.74, range 40 years). The youngest two patients at the time of surgery were 21 years old, the oldest 61. Postoperative follow-up was mean 63 months and median 57 months (SD = 41.01), approximately right-skewed distributed with a minimum follow-up of 12 months for 5 observations and a maximum of 180 months for 1 observation, corresponding to a range of 168 months.

### 2.2. MRI Acquisition

A 1.5-Tesla Magnetom Sola (Siemens Healthcare GmbH, Erlangen, Germany) and a 1.5-Tesla Magnetom Avanto (Siemens Healthcare GmbH, Erlangen, Germany) magnetic resonance scanner were used for the dedicated breast MRI examination, as nationwide and international Breast Imaging Working Groups recommended [16,17]. During the examination, the patients were in the prone position in an 18-channel breast dual coil (Breast 18, Siemens Healthcare GmbH, Erlangen, Germany and CP Breast Array Coil, Siemens Healthcare GmbH, Erlangen, Germany) with bilateral minimal lateral breast compression. The following examination sequences were scanned: STIR coronary, T2 TSE axial, and DWI axial. For the T1 FLASH 3D axial, 1 measurement before (native acquisition) and 6 measurements after administration of intravenous contrast agent (Clariscan or Prohance 0.1 mmol/kgKG) with a temporal resolution of 58 s at a voxel size of 0.7 × 0.7 × 2 mm were performed. The T1 images were evaluated by subtraction (the native T1 image is subtracted from the T1 with i.v. contrast). Table 2 shows the parameters and settings of the breast MRI protocols of both devices. Furthermore, T2 TSE (turbo spin echo) sequences of the transverse plane were considered and measured.

### 2.3. MRI Data Analysis

The following data were analyzed and recorded within the imaging data for each patient:

- Breast density;
- Preoperative breast volume;
- Postoperative breast volume;
- Skin flap thickness at 2, 3, 4, 6, 8, 9, 10, and 12 o’clock as well as retromamillary area;
- Any possible breast compression at each measurement point;
- Residual glandular tissue in retromamillary area;
- Residual glandular tissue at other location;
- Volume of the residual glandular tissue.

**Figure 1:**
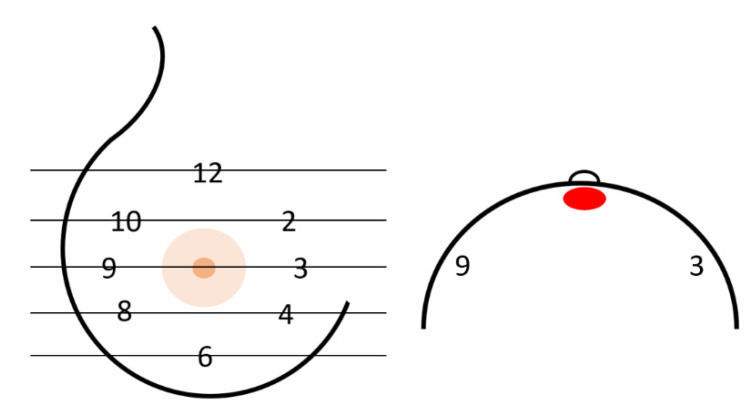
Frontal and craniocaudal schematic diagram of the breast with the measurement regions marked by time and red ellipse for the retromamillary measurement region. The lines in the left breast diagram are to represent the orientation in the MRI image.

**Figure 2:**
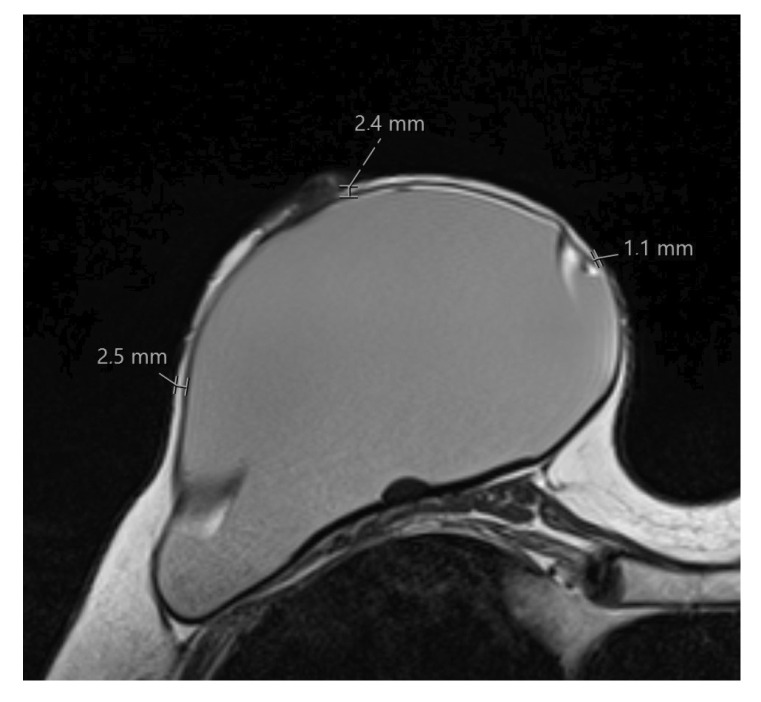
Skin flap measurement acquisition in T2-weighted image. Thickness measurements of retromamillary skin flap and at 3 and 9 o’clock. Measurements were performed using the integrated measurement tool of the Picture Archiving and Communication System (SECTRA AB, IDS7 Version 22.1, Linköping, Sweden).

**Figure 3:**
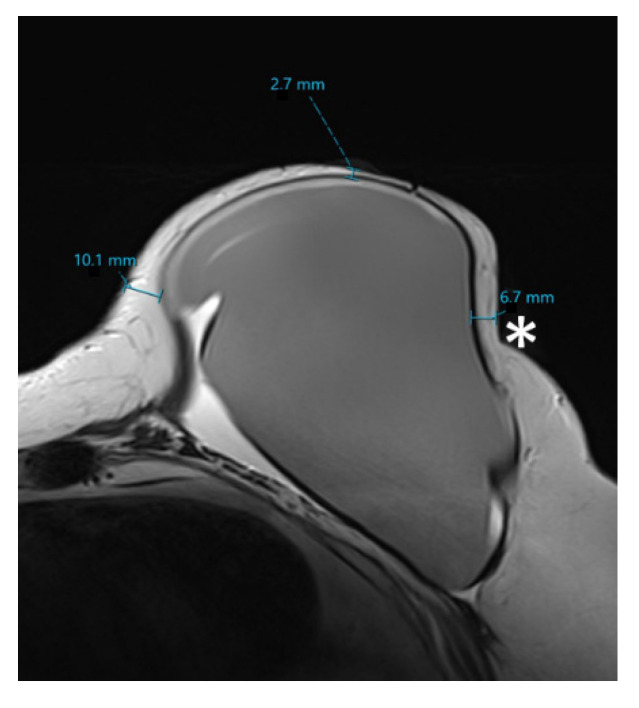
Example of compression (*) of the breast acquisition in T2-weighted image. Thickness measurements of retromamillary skin flap and at 3 and 9 o’clock. Measurements were performed using the integrated measurement tool of the Picture Archiving and Communication System (SECTRA AB, IDS7 Version 22.1, Linköping, Sweden).

The thickness of the remaining skin flap of each breast after mastectomy was measured with the measurement tool of the picture archiving and communication system (PACS, SECTRA AB, IDS7 Version 22.1, Linköping, Sweden). The defined measurement points were retromamillary, if the nipple was still present, and clockwise equally distributed in the 3/6/9 and 12 o’clock and 2/4/8 and 10 o’clock lines within the layers of the middle third of the breast, as shown in Figure 1 and Figure 2. Where MRI-related breast compression of the breast was visible, this was separately coded for each measurement point, as shown in Figure 3.

**Figure 4:**
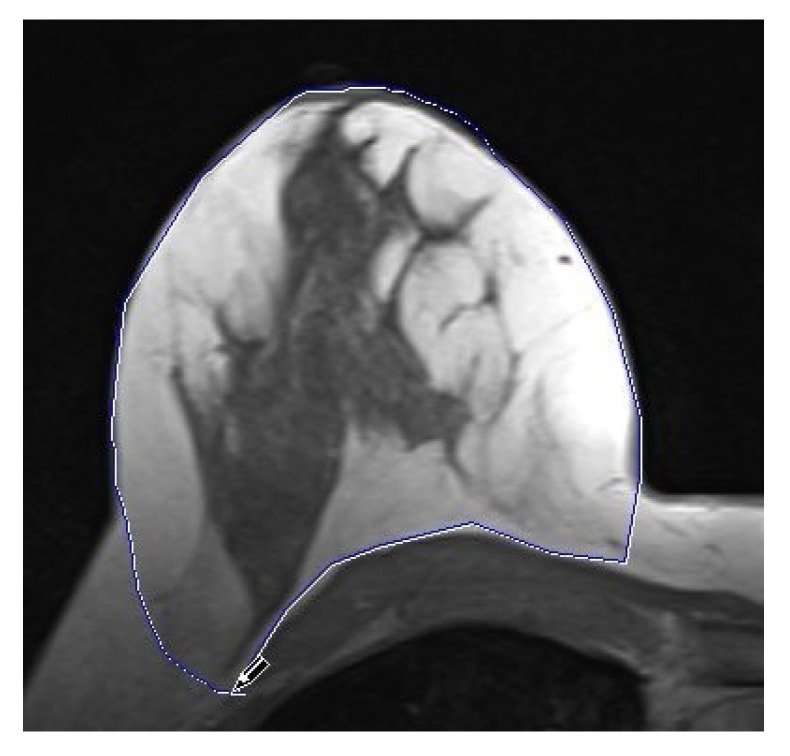
Preoperative volumetry of the whole breast T2-weighted image. The measurements were made using the image processing software Syngo.via (Siemens Healthcare GmbH, Erlangen, Germany).

**Figure 5:**
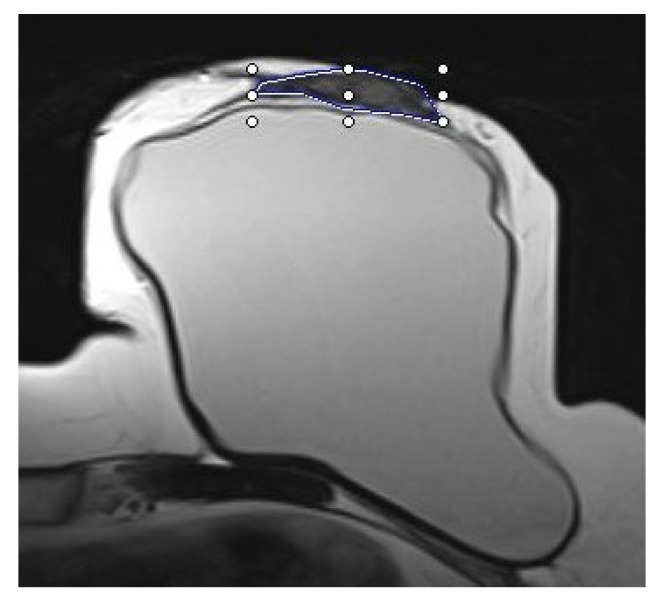
Volumetry of residual glandular tissue after risk-reducing mastectomy with implant T2-weighted image. The measurements were made using the image processing software Syngo.via (Siemens Healthcare GmbH, Erlangen, Germany).

In addition, each breast density before mastectomy and each volume of the breast before and after mastectomy were determined using the image processing software Syngo.via (Siemens Health-care GmbH, Erlangen, Germany), version 5.1. For this purpose, the breasts were marked on the transverse slices by traversing around the breast contour and displayed as corresponding areas, which were subsequently interpolated to a volume by the software. Ventrally, medially, and laterally, the natural mammary bulge with its lift from the thoracic contour was used as surface limitations, shown in Figure 4. The pectoralis major muscle was chosen as the dorsal anatomical limitation, as well as the bony thorax for the cranial images. For volumetry after mastectomy and implant insertion, the total breast volume was measured first and then the volume of the inserted implant was subtracted. If residual glandular tissue was found in any location, as Figure 5 shows, this was also recorded volumetrically.

### 2.4. Statistical Analysis

#### 2.4.1. Descriptive Statistics

The collected data were first analyzed descriptively (see Table 3). In total, 21 variables were metric (including 20 metric continuous and 1 metric discrete (breast density)) and 4 variables were categorically scaled (including 1 binary scaled (RRBM or RRCM), 3 nominal scaled (surgeon, surgical technique, and gene mutation), and 1 ordinal scaled (surgical experience)). For the continuous variables, minimum, maximum, median, mean, 5%, 25%, 75%, and 95% percentile, standard deviation, and range were determined; for the categorical variables, frequencies of the individual expressions were determined.

#### 2.4.2. Inductive Statistics

To detect possible associations, correlations were calculated for the patient- and breast-specific variables (unreported data). 

Subsequently, these possible associations were analyzed using univariate and multivariate linear OLS (ordinary least squares) regressions (see Table 4, Table 5 and Table 6). The dependent variable in the regressions was residual glandular tissue. Statistical significance was reported for the 10%, 5%, and 1% level.

In the first regressions (see Table 5), skin flap thickness measured in data collection acted as an independent variable after being operationalized in different ways: the maximum measured values were summarized in skin flap thickness max., analogously the smallest values were summarized in skin flap thickness min., the medians and means of all measured points were calculated in skin flap thickness median and skin flap thickness mean, the retromamillary recorded values were summarized in the skin flap thickness retromamillary area, and lastly the variable skin flap thickness mean without min./max. was calculated by summarizing mean values of skin flap thicknesses without the largest and smallest measured values in each case. The variable was calculated to correct for possible outliers within the measured values. 

These first OLS regressions (uni- and multivariate) were intended to test for a correlation of the independent variables with the amount of residual glandular tissue, to compare the significance of the differently operationalized variables, and lastly, to control for possible study-related bias in the measurement results (due to the breast compression). For this purpose, the skin flap thickness-specific variables were interacted with the variable coding for MRI-related breast compression.

In the following step, univariate OLS regressions were performed (see Table 6) to obtain an intuitive overview of the simple data correlations and to show the correlations of the independent variables with the dependent variable residual glandular tissue.

In the final step, we performed multivariate regression to comprehensively model the effects/correlations of the independent variables with the dependent variable (see Table 6).

All calculations and graphs were performed and generated using the Python 3 programming language together with the package “statsmodels”.

## 3. Results

A BRCA1 mutation was found in 49 patients (60.5%), of which 22 were operated by RRBM. BRCA2 mutation affected 24 (29.6%) patients, and of these 13 underwent bilateral mastectomy. One patient (1.2%) had both BRCA1 and 2 mutations. She belonged to the group of bilaterally operated patients. A PALB2 or CHEK2 mutation was present in three patients each (3.7%), and an ATM mutation in one case (1.2%). In two patients (2.5%), an informative genetic testing result was documented but the causative mutation was not. The mean preoperative breast volume was 857 mL (median 798 mL). The smallest volumes measured were 112 and 125 mL. They were attributable to a patient who underwent RRBM. The largest documented volumes were 2127 and 2063 mL, which also belong to one patient. The postoperative breast volume (total volume of each mamma minus the inserted implant volume) was 427 mL at the mean (median 417 mL). The maximum measured volume was 1078 mL, followed by 1040 mL. Both largest volumes were from one patient. The smallest measured volume was 69 mL, concerning two observations. Again, both measurements were from one patient. Residual retromamillary glandular tissue was found in 50 of the 117 cases (42.74%). None was found in 67 cases (57.26%). The largest volume of residual glandular parenchyma was 5.5 mL, and the smallest was 0.1 mL. Both volumes were measured once each. The mean value of residual glandular parenchyma (observations without residual glandular parenchyma excluded) was 1.58 mL, and the median was 1.3 mL. Table 3 shows a detailed description of all parameters of our study collective.

Table 3 reports all descriptive parameters. A total of 81 observations were recorded if they were patient-specific parameters and 117 for breast-specific parameters. Only 99 observations were recorded for the variable skin flap thickness retromamillary area (no nipple was left in the remaining observations). Mean and median values, standard deviation, smallest and largest recorded value, and 5% and 95% percentiles are given. Patient-related variables are the following: RRBM or RRCM, surgeon, surgical method, gene mutation (all categorial), age at the time of surgery in years, follow-up in months, and experience of the surgeon, all metric variables. The surgeon experience variable was calculated based on the number of surgeries he/she had already performed within the study cohort at the time of the surgery under consideration. Breast-related variables are breast volume pre- and postoperatively and skin flap diameters in millimeters, which were recorded clockwise as well as in the retromamillary area (if the nipple was preserved), including the following associated parameters: skin flap mean and median, skin flap minimum (min.) and maximum (max.), skin flap mean without min./max., and residual glandular tissue amount in milliliters (all metric). 

**Figure 6:**
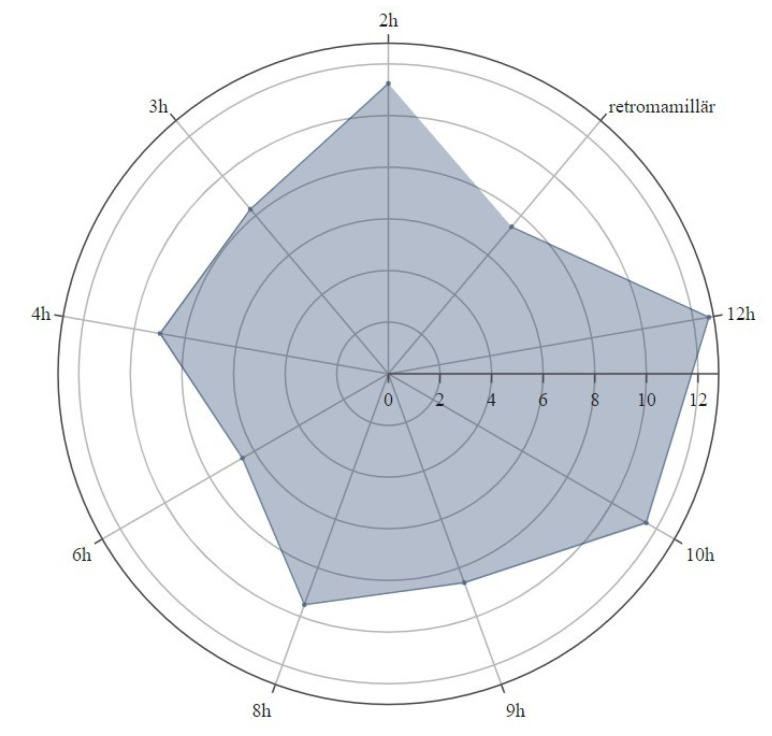
Skin flap measurements. Mean values of the skin flap diameters.

Figure 6 shows thickest skin flap measurements at 12 o’clock, followed by 10 and 2 o’clock. The remaining measurement points show thinner skin flaps, emphasized at 6 o’clock and in the retromamillary area.

### 3.1. Univariate Regressions

Table 4 shows the results of univariate OLS regression for the dependent variable residual glandular tissue amount. Skin flap thickness measured in data collection acted as an independent variable after being operationalized in different ways: the maximum measured values were summarized in skin flap thickness max., analogously the smallest values were summarized in skin flap thickness min., the medians and means of all measured points were calculated in skin flap thickness median and skin flap thickness mean, the retromamillary recorded values were summarized in skin flap thickness of the retromamillary area, and lastly the variable skin flap thickness mean without min./max. was calculated by summarizing mean values of skin flap thicknesses without the largest and smallest measured values in each case. The variable was calculated to correct for possible outliers within the measured values. The regression was intended to test for a correlation of the independent variables with the amount of residual glandular tissue to compare the significance of the differently operationalized variables, and lastly, to control for possible study-related bias in the measurement results. For this purpose, the skin flap thickness-specific variables were interacted with the variable coding for MRI-related breast compression. A positive effect of all investigated independent variables with the amount of residual glandular tissue was shown. Skin flap thickness max., skin flap thickness median & skin flap thickness mean without min./max. are significant at the 5% percentile, and skin flap thickness min., skin flap thickness median, and skin flap thickness retromamillary values at the 1% percentile. No interaction of skin flap thickness with measured breast compression was detected in any case, seen in columns 2, 4, 6, 8, and 10. Model performance (R2) is moderate, with variables explaining only a maximum of 14.6% of the variance in measured values, seen in column 12 for the skin flap thickness retromammary variable.

Table 5 shows the results of univariate regression for the dependent variable residual glandular tissue amount. A significant effect at the 5% percentile can be shown for the independent variables surgeon experience and ln(surgeon experience). Logarithmization was used to spread small numerical values (at the beginning of data collection, where surgeon experience was always low in our cohort) and to compress large numerical values. This has the effect of reducing the influence of extreme observations on the estimate or making skewed distributions more symmetric. In our sample, however, logarithmization does not change the effect of the studied variable surgeon experience. The greater the experience of the surgeon performing the procedure, the less residual glandular tissue is detectable. This is significant at the 1% percentile for the variable surgical method, including the derived variable nipple-sparing mastectomy (NSM) with inframammary fold incision, which compares the surgical method with the other surgical methods performed. The variable is non-metric, which complicates the interpretation of the regression. For the surgical method, numbering was conducted in the following manner: NSM with inframammary fold incision was numbered 1, followed by NSM with inverted T incision, skin sparing mastectomy (SSM) using spindle-shaped incision, and SSM with inverted T incision. For the variable NSM with inframammary fold incision, all cases with the corresponding surgical method were marked with 1, and those cases with other surgical methods were marked with 0. Thus, the negative sign of the coefficient of the surgical method indicates a larger amount of residual glandular after NSM with inframammary fold incision, compared to the other techniques used. No significant relationship can be shown for the independent variables age at the time of surgery, surgical indication, breast density, and breast volume (pre- and postoperative). Model performance is rather low for the regression presented, as *R*^2^ can only explain a maximum of 9.3% of the variance of the measured values, seen in Table 6, column 9 for the independent variable NSM with inframammary fold incision.

### 3.2. Multivariate Regressions

Table 6 shows the results of the multivariate regressions for the variable residual glandular tissue amount. It can be shown that higher breast density leads to more residual glandular tissue. This effect is robust across all specifications and significant at the 1% percentile. Skin flap thickness also shows a positive relationship to residual glandular tissue amount, significant at the 1% percentile, the effect being the same for the variables skin flap thickness retromamillary value and skin flap thickness mean. Other skin-flap-thickness-specific variables were not used for the multivariate regressions because it has already been shown in the univariate regression that similar relationships exist with the dependent variable residual glandular tissue volume. The interaction with the compression variable shows that study-related breast compression does not affect the results. Multivariate analysis also demonstrates that NSM with an inframammary fold incision results in more residual glandular tissue compared with the other surgical procedures (significant at the 1% percentile). Surgeon experience actually also shows a robust effect. In the regressions where it is not significant, the *p*-value is usually only slightly above 10%. *R*^2^ is high in the final regressions (up to 32.2%). This shows the good model performance.

## 4. Discussion

For women of the high-risk population, the cumulative risk of developing breast cancer by age 80 is 72% for BRCA1 and 69% for BRCA2 carriers. The breast cancer is more often bilateral and multifocal in patients with a BRCA mutation [18]. Very effective preventive measures, including chemoprevention, intensified monitoring, and preventive surgery, remain effective, but for some measures, such as bilateral prophylactic mastectomy, the overall survival benefit is not yet clearly defined [19,20]. It remains clear that bilateral risk-reducing mastectomy achieves the greatest risk reduction in developing breast cancer by about 90% [21]. Nevertheless, in all forms of mastectomy residual glandular tissue and therefore the risk of developing breast cancer remains [11]. It is important to find appropriate measurement parameters that make this risk tangible. For this reason, we highlight the factors influencing residual glandular tissue in our study.

### 4.1. Amount of Residual Glandular Tissue

The largest amount of residual glandular tissue recorded volumetrically in our study is 5.5 mL, and the smallest is 0.1 mL. Both volumes were measured once each. The mean value of postoperative residual glandular tissue is 1.58 mL, and the median is 1.3 mL. In 94% of the cases in which residual glandular tissue was detected, the proportion of residual glandular tissue in the preoperative breast volume of the patients was (mostly far) below 1%. Only in three cases (6%) did the amount of residual glandular tissue exceed 1% of the total volume. When comparing our study results with those of other studies, the amount of residual glandular tissue in our cohort is very low. Volumes of a maximum of 5.5 mL and a mean value of 1.58 mL, which in almost all cases represent a proportion of less than 1% of the total breast volume, are to be considered minimal. This is also supported by other studies: The residual glandular tissue of the nipple areolar complex (NAC) in the studies by Baltzer et al. accounted for only up to 1.2% of the total glandular tissue [22]. Grinstein et al. describe average amounts of postoperative residual glandular tissue of 2–3 mL [23]. Papassotiropoulos et al. described significantly larger amounts: a median of 7.1% of the total glandular tissue was found postoperatively [11]. There are also clear parallels to the studies by Grinstein et al. with regard to localization: there, too, residual glandular tissue was found in the retromamillary area in 94% of the detected cases [23]. This corresponds exactly to our results.

### 4.2. Frequency of Residual Glandular Tissue

In our cohort, residual glandular tissue was detected first and foremost in the retromamillary area in 50 breasts (42.74%). In two cases, in addition to the retromamillary area, residual glandular tissue was detected at another site. Accordingly, all cases of residual glandular tissue occurred after nipple-sparing mastectomies (NSM). The existing literature shows a heterogeneous picture in the frequency of residual glandular tissue present postoperatively. In several studies, residual glandular tissue was found in 6–76.2% of the breasts examined after SSM [24,25]. Papassotiropoulos et al. found residual glandular tissue in 51.3% of cases [11]. Comparing these data with those of our study, special attention should be paid to the surgical technique separately: In our study, in 94% of all cases in which residual glandular tissue was detected, it was found in the retromamillary area. Excluding all cases of retromamillary residual glandular tissue (to simulate analogous conditions to the study results for residual glandular tissue after SSM), residual glandular tissue was found in only 1.71%. Giannotti et al. found residual glandular tissue outside the NAC in 29.9% of cases after subcutaneous mastectomy, Grinstein et al. without exclusion of the NAC in 37.9%, and both studies considered both risk-reducing and therapeutic mastectomies [23,26]. However, other studies of residual glandular tissue after mastectomies have shown that residual glandular tissue is present postoperatively in 5% to 100% of all mastectomized patients [11,12]. A recent meta-analysis described the variables surgical method, surgical indication, as well as surgeon experience as significant influencing factors for residual glandular tissue [27]. In our study, consistent results were obtained with regard to postoperative residual glandular tissue in the univariate and multivariate analyses, although the multivariate analysis was able to identify a larger number of influencing factors overall, which are described in more detail below. 

### 4.3. Influence Factor Skin Flap Thickness

One of the influencing factors is the postoperatively remaining skin flap thickness. It had a significant effect in all analyses. This was true both for the sum of all measurement points and in isolation for the skin flap thickness retromamillary value, which was considered separately because of its relation to most of the detected residual glandular tissue. The current literature supports the results we have shown regarding the significant effect of skin flap diameter on postoperative residual glandular tissue [23,26]. Similarly, across studies, more residual glandular tissue can be found behind the NAC [23,28]. 

### 4.4. Influence Factor Surgeon Experience

The analyzed variable surgeon experience had a negative effect on postoperative residual glandular tissue (significant at the 5% level) in univariate analysis; in multivariate analysis, the effect was significant at the 5% or 10% level in most regressions. In a few regressions, the value was just above 10%, but in the aggregate, a robust effect can be seen. Kaidar et al. were also able to show an influence of surgeon experience, analogous to our results [28]. 

### 4.5. Influence Factor Surgical Method

For the variable surgical method, we investigated first within the multivariate analysis whether an effect on postoperative residual glandular tissue could be shown by performing an NSM with an inframammary fold incision in contrast to the other surgical methods performed. A positive effect was shown with more residual glandular tissue in favor of NSM with an inframammary fold incision. Since no residual glandular tissue was detectable after SSM in any observation in our study, the surgical method has a strong negative effect on residual glandular tissue in the univariate regression. Because of the unambiguous data, we did not further test the relationship in the multivariate analyses. Analogous to our results, Papassotiropoulos et al. also showed significant effects of the chosen surgical method [11]. 

### 4.6. Influence Factor Breast Density

While the variable breast density failed to show an effect on the amount of residual glandular tissue present postoperatively at the univariate analysis, the effect was significant in multivariate analysis. Since the multivariate analysis provides more specific results and eliminates influences of other variables, an existing effect of the variable can be assumed. The higher the patient’s breast tissue density, the greater the risk for residual glandular tissue present postoperatively. According to our research, there are no publications that have addressed the relationship between breast density and residual glandular breast tissue after risk-reducing mastectomy. This is a point which needs to be further investigated in future studies.

### 4.7. Influence Factor Breast Volume

With regard to the influencing factor breast volume, in the univariate analyses, both preoperative and postoperative breast volume were examined. Because all analyses showed equal effects for preoperative and postoperative breast volume, only preoperative volume was used for the multivariate analyses to avoid colinear variables. Preoperative volume was used because, on the one hand, postoperative volume is already approximated in the analysis by the skin flap variable, and, on the other hand, preoperative volume can function as a component of preoperative risk assessment. Multivariate analysis shows a negative effect of preoperative breast volume on possible residual glandular tissue: the larger the volume, the smaller the probability of postoperative residual glandular tissue. 

### 4.8. Variables with No Effect on Residual Glandular Tissue

The variables age and surgical indication (whether an RRBM or RRCM was performed) had no effect on residual glandular tissue. Some other studies came to contrary results with significant effects of the variables on postoperative residual glandular tissue [23,28,29]. Moreover, no influence was found by minimal MRI-related compression of the breast during breast MRI in our study. This correlation is not causal, since compression during postoperative MRI does not lead to more or less residual glandular tissue but shows that the compression does not distort the measurement result and speaks for the validity of the measured values. 

One of the limitations of the study is the monocentric design. With a multicenter study, it would be possible to analyze the covariates in more detail and thus to assess the effects of the independent variables more accurately. This would improve the probability calculations of the residual risk of breast cancer after risk-reducing mastectomy. Another limitation is that only surgeons with a high level of experience were represented in our study. For further studies, attention should be paid to the heterogeneity of the surgeons with regard to the level of experience. Another limitation is that fewer postoperative breast MRIs were performed in the early stages of the retrospective study (2006), so many patients were excluded.

## 5. Conclusions

For the independent variables breast density, skin flap thickness, surgical method, preoperative breast volume, and surgeon experience, different effects on residual glandular tissue could be shown, so that these should be considered in future surgical procedures preoperatively as well as postoperatively. Breast MRI has proven to be a suitable method for analyzing the skin flap as well as residual glandular tissue. Compared with other studies, the amount of residual glandular tissue found after risk-reducing mastectomy is particularly low in our study. One possible explanation for this is that the medical staff has interdisciplinary training, e.g., all surgeons performing senological surgery are also extensively trained in diagnostic methods and perform some of these themselves. However, the data of our retrospective study come from only 1 of 24 German Consortium sites for Hereditary Breast and Ovarian Cancer (GCHBO). In order to validate our results in the best possible way, an interdisciplinary, prospective randomized study would be desirable.

## Figures and Tables

**Table 1:** Study cohort description.

Numbers of patients	81
Numbers of breasts	117
Age at time of surgery (mean)	40.12 years
Follow up (mean)	63 months
Mutation types	
BRCA 1 mutation carriers	49
BRCA 2 mutation carriers	24
Carriers of other or unknown mutation types	9
Breast volume (mean)	
preoperative	857 mL
postoperative	427 mL
Residual glandular tissue	
Detection frequency	50
Amount (mean), when detected	1.58 mL

Abbreviations: BRCA, breast cancer associated gene.

**Table 2:** Parameters and settings of the selected T2 TSE MRI sequence of both used devices.

Parameter	Magnetom Sola	Magnetom Avanto
TR (ms)	3670	5800
TE (ms)	73	101
fov (Pixel)	340 × 340	340 × 340
voxel (mm^3^)	0.7 × 0.7 × 2	0.8 × 0.7 × 2.5
slice thickness (mm)	3	4
slice gap (mm)	3.3	5
number of slices	58	33
coils	mammary dual coil	mammary dual coil
	(Breast 18 Tim coil)	(CP Brust Array)
contrast agent	Clariscan	Prohance
dose of contrast agent (mmol/kgKG)	0.1	0.1
time resolution (s)	58	58

Listed are all relevant parameters of the sequence selected for data analysis for the 1.5 Tesla Magnetom Sola and the 1.5 Tesla Magnetom Avanto Magnetic Resonance Tomograph. Abbreviations: TR, repetition time; TE, echo time; fov, field of view.

**Table 3:** Descriptive parameters.

	Mean	SD	Min.	5th	50th	95th	Max.
Age	40.12	8.74	21.00	26.00	40.00	54.00	61.00
Follow-Up	61.30	39.67	12.00	21.00	52.00	157.00	180.00
Surgeon	1.78	1.44	0.00				6.00
Surgical experience	16.32	12.65	1.00	1.00	14.00	40.00	44.00
RRBM or RRCM	0.56	0.50	0.00				1.00
Surgical technique	1.85	1.33	1.00				5.00
Gene mutation	1.49 *	0.82	0.00				6.00
Preop. breast vol.	856.86	455.84	112.00	231.80	798.00	1691.20	2127
Postop. breast vol.	426.71	218.84	69.00	125.00	417.00	790.00	1078
Breast density	2.82	0.87	1.00	1.00	3.00	4.00	4.00
Skin flap 2 h	11.24	6.90	1.00	2.80	10.00	23.20	36
Skin flap 3 h	8.32	5.39	1.00	2.00	7.00	17.40	26
Skin flap 4 h	8.98	6.11	1.00	1.80	8.00	20.20	29
Skin flap 6 h	6.53	5.29	1.00	2.00	4.00	17.20	24
Skin flap 8 h	9.51	7.18	1.00	2.00	7.00	23.40	31
Skin flap 9 h	8.61	5.68	11.00	2.00	7.84	20.00	25
Skin flap 10 h	11.54	6.34	1.00	3.00	10.00	24.00	30
Skin flap 12 h	12.62	8.37	2.00	2.64	11.00	27.00	40
Skin flap retromamillary	7.43	6.01	1.00	2.00	5.00	19.60	34
Skin flap max.	17.19	7.72	3.00	7.00	16.00	30.20	40
Skin flap median	10.30	5.45	2.00	3.00	9.00	21.10	25.00
Skin flap min.	3.97	3.03	1.00	1.00	3.00	9.40	17.00
Skin flap mean	17.70	8.88	4.83	6.13	16.00	33.37	45.83
Skin flap mean without min./max.	6.05	3.09	1.71	2.07	5.43	11.50	15.85
Residual glandular tissue amount	0.65	1.01	0.00	0.00	1.20	2.62	5.50

* For the Gene mutation variable, there is a peculiarity that affects the calculations: one patient has both a BRCA1 and −2 gene mutation. The BRCA1 mutation was set as the basis for the values shown in the table. The following values result if her BRCA2 mutation is used as the basis for the calculation instead: mean = 1.50, standard deviation = 0.82. Abbreviations: SD, standard deviation; min., minimum; max., maximum; RRBM, risk-reducing bilateral mastectomy; RRCM, risk-reducing contralateral mastectomy.

**Table 4:** OLS regression—residual glandular tissue amount.

	1	2	3	4	5	6	7	8	9	10	11	12
Intercept	0.2230	0.2029	0.3319 *	0.3279	0.2948 *	0.3097 *	0.2164	0.2111	0.2179	0.2123	0.2855 *	0.3049 *
	(0.2258)	(0.2600)	(0.1993)	(0.2282)	(0.1497)	(0.1710)	(0.2055)	(0.2362)	(0.2022)	(0.2321)	(0.1589)	(0.1803)
Breast compression		0.4140		0.2290		−0.0474		0.2862		0.2687		0.1094
		(0.6334)		(0.5228)		(0.5676)		(0.6060)		(0.5852)		(0.4159)
Skin Flap max.	0.0248 **	0.0268 **										
	(0.0120)	(0.0131)										
Skin Flap max.: Compression		−0.0394										
		(0.0445)										
Skin flap median			0.0308 *	0.0333 *								
			(0.0171)	(0.0187)								
Skin flap median: Compression				−0.0462								
				(0.0579)								
Skin flap min.					0.0892 ***	0.0876 ***						
					(0.0300)	(0.0317)						
Skin flap min.: Compression						0.0001						
						(0.2241)						
Skin flap mean							0.0244 **	0.0254 **				
							(0.0104)	(0.0113)				
Skin flap mean: Compression								−0.0286				
								(0.0429)				
Skin flap mean ex min./max.									0.0714 **	0.0744 **		
									(0.0298)	(0.0324)		
Skin flap mean ex min./max.: Compression										−0.0796		
										(0.1200)		
Skin flap retromamillary											0.0625 ***	0.0652 ***
											(0.0164)	(0.0175)
Skin flap retromamillary: Compression												−0.0564
												(0.0584)
R^2^	0.0359	0.0439	0.0274	0.0354	0.0714	0.0717	0.0460	0.0506	0.0475	0.0521	0.1300	0.1445
Adj. R^2^	0.0275	0.0185	0.0189	0.0098	0.0634	0.0471	0.0377	0.0253	0.0392	0.0269	0.1211	0.1175
N	117	117	117	117	117	117	117	117	117	117	99	99

The table shows the results of univariate and multivariate OLS regression (linear regression) for the dependent variable residual glandular tissue amount. Standard errors are indicated in parentheses; *, **, and *** indicate statistical significance for the 10%, 5%, and 1% levels. Abbreviations: Const., regression constant; *R*^2^, coefficient of determination; Adj. *R*^2^, adjusted coefficient of determination; N, number of observations; min., minimum; max., maximum.

**Table 5:** Univariate regression—residual glandular tissue amount.

	1	2	3	4	5	6	7	8	9
Intercept	7.3553 **	16.7016 ***	35.1283 ***	16.8605 ***	3.2237 ***	5.9272 ***	27.0457 ***	15.6313 ***	22.7507 ***
	−37,021	−10,390	−22,151	−12,077	(0.9751)	−12,531	−20,650	−13,805	−11,900
Age	0.2650 ***								
	(0.0925)								
RRBM o. RRCM		26,334							
		−16,753							
Breast density			−6.2014 ***						
			(0.7512)						
Breast side				15,858					
				−16,458					
Postop. breast volume					0.0340 ***				
					(0.0020)				
Preop. breast volume						0.0137 ***			
						(0.0013)			
ln(surgeon experience)							−3.7548 ***		
							(0.7741)		
Surgical method Inframammary fold incision NSM								1.1606 *	
								(0.6222)	
Surgeon experience									−0.3045 ***
									(0.0565)
R^2^	0.0666	0.0210	0.3762	0.0080	0.7081	0.4982	0.1698	0.0294	0.2015
Adj. R^2^	0.0585	0.0125	0.3707	−0.0006	0.7056	0.4938	0.1626	0.0209	0.1945
N	117	117	115	117	117	115	117	117	117

The table shows additional results of univariate OLS regression (linear regression) for the dependent variable residual glandular tissue amount. Standard errors are indicated in parentheses; *, **, and *** indicate statistical significance for the 10%, 5%, and 1% levels. Abbreviations: Const., regression constant; *R*^2^, coefficient of determination; Adj. *R*^2^, adjusted coefficient of determination; N, number of observations; RRBM, risk-reducing bilateral mastectomy; RRCM, risk-reducing contralateral mastectomy; Postop., postoperative; Preop., preoperative; NSM, nipple-sparing mastectomy; ln, logarithmization.

**Table 6:** Multivariate regression—residual glandular tissue amount.

	1	2	3	4	5	6	7	8	9	10
Intercept	1.2756 ***	−0.8475	1.4773 ***	−0.6011	1.6765 ***	−0.4200	1.6708 ***	−0.4183	1.0220 *	−0.7400
	(0.4321)	(0.8042)	(0.4804)	(0.8057)	(0.5175)	(0.8493)	(0.5337)	(0.8533)	(0.5335)	(0.7827)
Age	−0.0254 **	−0.0139	−0.0256 **	−0.0133	−0.0264 **	−0.0141	−0.0263 **	−0.0142	−0.0300 ***	−0.0196 *
	(0.0112)	(0.0121)	(0.0112)	(0.0119)	(0.0113)	(0.0120)	(0.0114)	(0.0121)	(0.0108)	(0.0111)
RRBM o. RRCM	0.0905	0.0172	0.0936	0.0159	0.0601	−0.0045	0.0600	−0.0048	0.2166	0.1614
	(0.1936)	(0.1971)	(0.1937)	(0.1948)	(0.1963)	(0.1976)	(0.1973)	(0.1985)	(0.1910)	(0.1848)
Breast density		0.4012 ***		0.4335 ***		0.4234 ***		0.4268 ***		0.3649 ***
		(0.1381)		(0.1376)		(0.1387)		(0.1411)		(0.1296)
Breast side	−0.0620	−0.0715	−0.0535	−0.0484	−0.0720	−0.0618	−0.0724	−0.0604	−0.0367	−0.0030
	(0.1783)	(0.1796)	(0.1786)	(0.1780)	(0.1795)	(0.1795)	(0.1805)	(0.1805)	(0.1707)	(0.1654)
Preop. breast volume	−0.0009 ***		−0.0008 ***		−0.0008 ***		−0.0008 ***		−0.0002	
	(0.0003)		(0.0003)		(0.0003)		(0.0003)		(0.0003)	
Breast compression					−0.2598	−0.1743	−0.2344	−0.2559	−0.4893	−0.6163
					(0.2516)	(0.2527)	(0.5979)	(0.5978)	(0.5689)	(0.5517)
Skin flap mean	0.0640 ***	0.0537 ***	0.0574 ***	0.0460 ***	0.0536 ***	0.0428 ***	0.0537 ***	0.0425 ***	0.0393 **	0.0493 ***
	(0.0144)	(0.0129)	(0.0160)	(0.0134)	(0.0164)	(0.0142)	(0.0166)	(0.0144)	(0.0162)	(0.0132)
Skin flap mean: Compression							−0.0020	0.0063	0.0121	0.0271
							(0.0416)	(0.0419)	(0.0395)	(0.0385)
Inframammary fold incision-NSM									0.8279 ***	0.8553 ***
									(0.2227)	(0.1826)
Surgeon experience			−0.0074	−0.0142 *	−0.0092	−0.0154 *	−0.0092	−0.0155 *	−0.0136 *	−0.0169 **
			(0.0077)	(0.0076)	(0.0079)	(0.0078)	(0.0079)	(0.0079)	(0.0076)	(0.0072)
R^2^	0.1715	0.1582	0.1785	0.1848	0.1866	0.1884	0.1867	0.1886	0.2813	0.3289
Adj. R^2^	0.1335	0.1196	0.1329	0.1395	0.1334	0.1353	0.1253	0.1274	0.2197	0.2714
N	115	115	115	115	115	115	115	115	115	115

The table shows results of the multivariate regression for the dependent variable residual glandular tissue amount. Standard errors are indicated in parentheses; *, **, and *** indicate statistical significance for the 10%, 5%, and 1% levels. Abbreviations: Const., regression constant; *R*^2^, coefficient of determination; Adj. *R*^2^, adjusted coefficient of determination; N, number of observations; RRBM, risk-reducing bilateral mastectomy; RRCM, risk-reducing contralateral mastectomy; Preop., preoperative; NSM, nipple-sparing mastectomy.

## Data Availability

The datasets used and/or analyzed during the current study are available from the corresponding author on reasonable request.
